# Early tangential excision debulking after free latissimus dorsi flap reconstruction for soft tissue defects: presentation of three cases

**DOI:** 10.1080/23320885.2022.2128358

**Published:** 2022-10-06

**Authors:** Hiroko Murakami, Kazuo Sato, Yuta Izawa, Tatsuhiko Muraoka, Yoshihiko Tsuchida

**Affiliations:** a Department of Orthopedic Trauma Center, Sapporo Higashi Tokushukai Hospital, Sapporo, Japan; b Department of Orthopedic Trauma Center, Shonan Kamakura General Hospital, Kamakura, Japan

**Keywords:** Debulking surgical procedures, esthetics, free tissue flaps, tangential excision

## Abstract

Bulkiness is patients’ major complaint after free latissimus dorsi (LD) flap. We performed tangential excision debulking at 6–13 days following free LD flap in three patients. No flap necrosis or major complications occurred. Tangential excision debulking during the early phase after free LD flap might be safe and reliable.

## Introduction

Free flaps have been used for the coverage of soft tissue defects. Good results can be achieved using various flaps, such as latissimus dorsi (LD), anterolateral thigh, and groin flaps. In particular, free LD flap is versatile in covering large soft tissue defects. However, bulky appearance is the major complaint of patients after free LD flap reconstruction. This bulky appearance results in esthetic problems and functional issues, such as unfitting shoes or sleeves. Several surgical methods to solve these problems have been reported, one of which is primary thinning after flap elevation. However, this method may injure blood vessels and, thus, risk flap survival. The staged debulking procedure might be safe and can lead to good outcomes. However, this method is time-consuming and costly owing to multiple operations.

Although several reports of one-stage debulking procedure have revealed good results, debulking was performed at least 3–4 months, sometimes years, after free flap reconstruction.

Herein, we report early tangential excision debulking after free LD flap, which achieved satisfactory results esthetically and functionally in the early stages. Using this method, the debulking procedure could be completed at 1 month after flap reconstruction.

## Case report

### Patient 1

**Figure 1.**
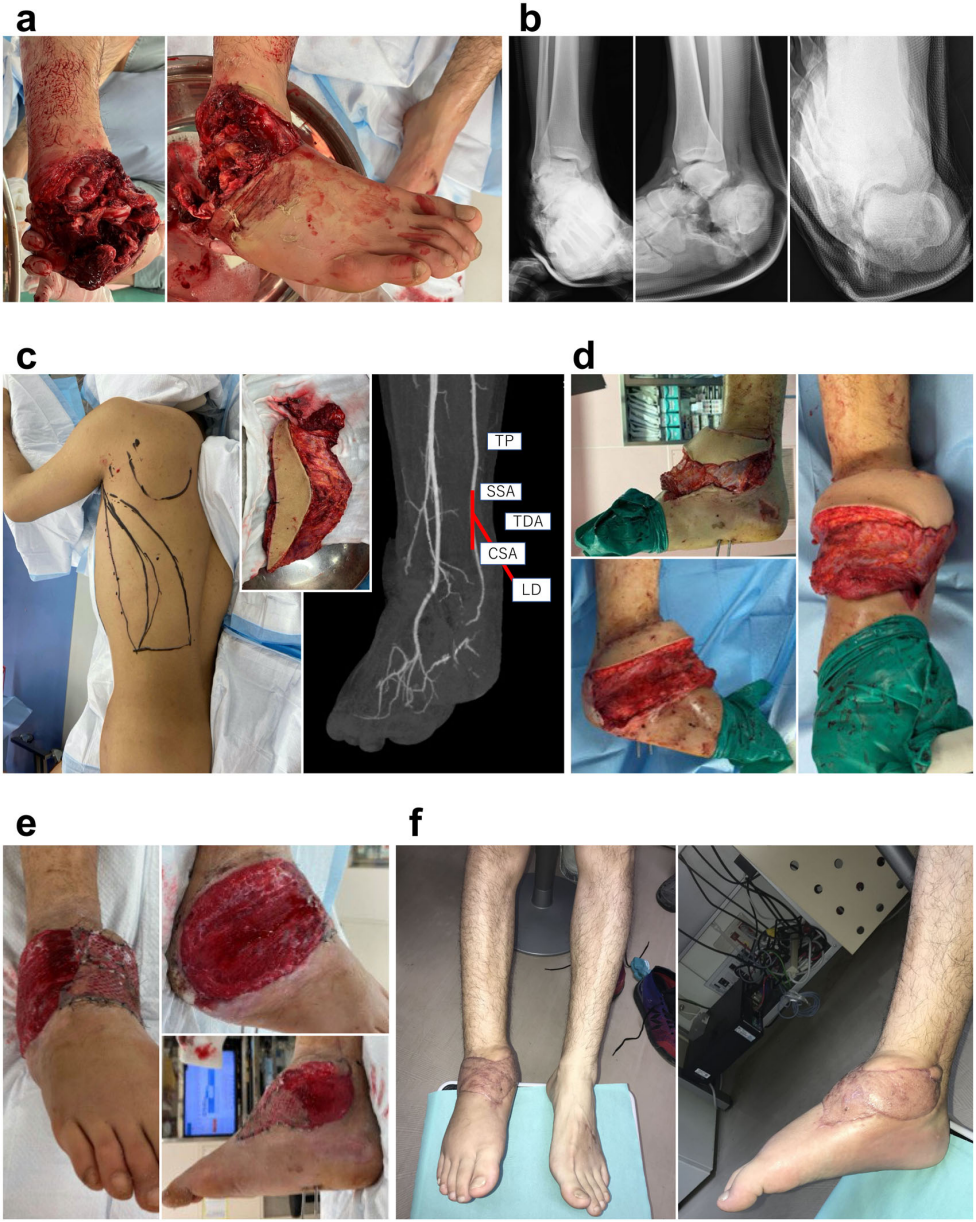
Patient 1. (A) Upon arrival at the emergency room, the blood circulation in the right foot was poor. (B) Multiple fractures and dislocations were observed after performing radiographic examination. (C) The recipient artery was interposed with T portion and the flow was sutured through anastomosis to the TP artery. (D) On day 9, free LD flap was performed. Abbreviation: (E) After the third tangential excision (4 weeks after LD flap). (F) Appearance at 3.5 months after injury. TP: tibialis posterior artery; SSA: subscapular artery; CSA: circumflex scapular artery; TDA: thoracodorsal artery; LD: latissimus dorsi.

A 28-year-old man presented with a crush injury to the right foot after a heavy machine fell on his leg. Upon his arrival at the emergency room, his right foot was ischemic (Figure 1(A,B)). The patient was immediately transferred to the operating theater and underwent debridement and temporary K-wire fixation. Although there were multiple tarsal bone fractures and soft tissue defects, the patient’s main arteries and nerves were preserved. After several debridement procedures and bone fixation with plates, the soft tissue defect was 30 × 15 cm in size. On day 9 after injury, reconstruction was performed using free LD flap (Figure 1(C,D)). The donor vessel was interposed with T portion to the tibialis posterior (TP) artery. The thoracodorsal vein (TDV) was sutured to the accompanying vein of TP by end-to-end anastomosis. At 12 days after free flap reconstruction, the first tangential excision was performed. Following three tangential excisions in total, a split-thickness skin graft was performed using free flap skin. In this procedure, the skin was harvested from the ipsilateral thigh 4 weeks after free LD flap (Figure 1(E)). The grafted skin was completely obtained without interfering with the flap blood circulation.

The patient had to wear a non-weight-bearing brace for 3 months owing to concomitant skeletal injury (Figure 1(F)). He could walk normally with regular shoes without pain after he was allowed to remove the brace 3 months post-injury.

### Patient 2

**Figure 2.**
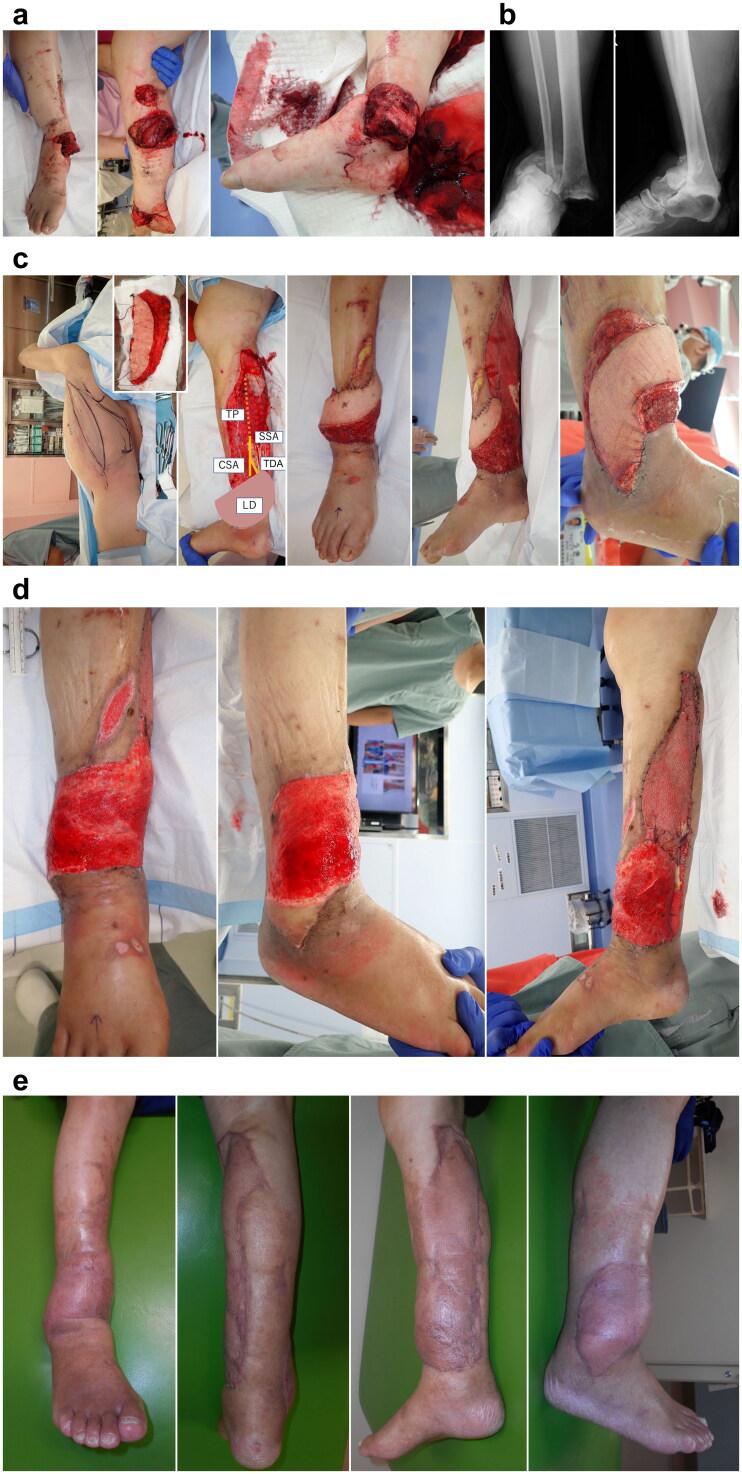
(A) Appearance on his arrival. (B) Radiographic findings show ankle dislocation fracture. (C) Free LD flap was performed at 4 weeks after injury. The recipient artery was interposed with T portion and the flow was sutured through anastomosis to the TP artery. (D) The fourth tangential excision was performed at 3.5 weeks after free LD flap. (E) Findings at 3 months after free LD flap. TP: tibialis posterior artery; SSA: subscapular artery; CSA: circumflex scapular artery; TDA: thoracodorsal artery; LD: latissimus dorsi; LD: latissimus dorsi TDA: thoracodorsal artery; UA: ulnar artery.

A 66-year-old man was caught in a cultivator machine. It took more than 3 h to rescue him from the machine. Upon hospitalization, the patient was in a shock state due to blood loss. He was transferred to our hospital 7 h after his injury (Figure 2(A,B)). Although we performed emergency debridement thoroughly, the wound was contaminated, and an infection developed. Several debridement procedures were needed because of wound infection before bone fixation. After the infection subsided, soft tissue reconstruction was performed using free LD flap at 4 weeks after injury (Figure 2(C)). The subscapular artery (SSA) with the circumflex scapular artery (CSA) were interposed to anastomose between the transected TP. The TDV was end-to-end anastomosed with the accompanying vein of TP. On day 6 after flap reconstruction, the first tangential excision was performed, and several tangential excisions were repeated using a razor blade, arthroscopic shaver, and hydrosurgery system (Figure 2(D)). Full-thickness skin graft (FTSG) was performed at 4 weeks after free flap reconstruction. The wound healed without complications. The patient could walk with regular shoes without assistance (Figure 2(E)).

### Patient 3

**Figure 3.**
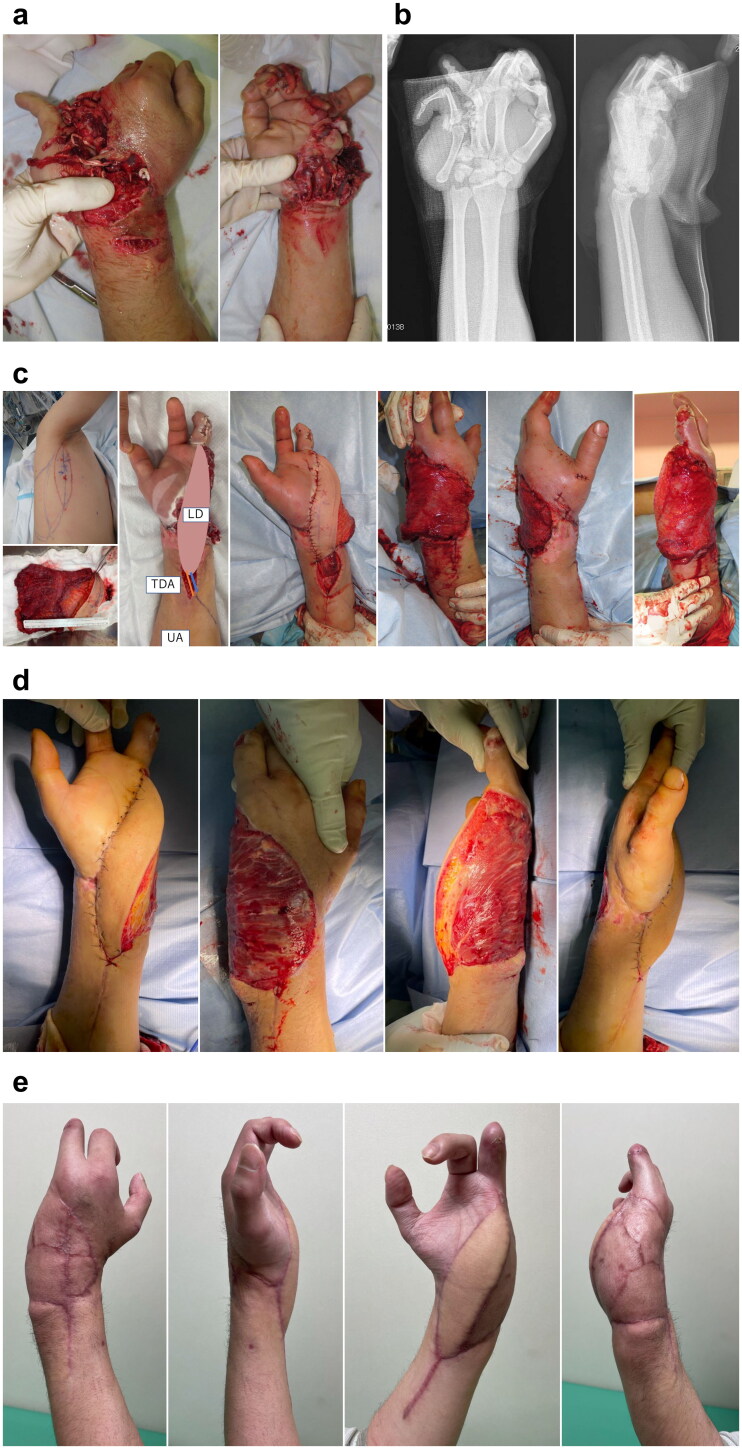
(A) Appearance on his arrival. (B) Radiographic examination on his arrival. (C) Free LD flap was performed at 1 week after injury. The recipient artery was sutured end-to-end to the ulnar artery. (D) After the third tangential excision, (E) Findings at 3 months after free LD flap. LD: latissimus dorsi.

A 33-year-old man sustained a crush injury to the right hand. His arm was caught in a conveyor belt (Figure 3(A,B)). The patient was transferred to the operating theater, and revascularization was performed with debridement and pinning due to hand ischemia. A free LD flap was performed to cover the 18 × 15-m soft tissue defect at 1 week after revascularization, waiting until blood circulation stabilized (Figure 3(C)). The TDA and the ulnar artery (UA) were end-to-end anastomosed. The TDV and the subcutaneous vein were sutured with vein graft. On day 13 after flap reconstruction, the first tangential excision was performed. Three tangential excision procedures were performed in total followed by FTSG 4 weeks after flap reconstruction (Figure 3(D)). Then, the patient could wear regular clothes and use his hand (Figure 3(E)).

## Discussion

Free flap reconstruction for soft tissue defects after severe extremity trauma can provide good results, and sometimes, this is the only way to avoid limb amputation. However, the bulkiness of the flap is a major cause of unsatisfactory results. Patients complain because they cannot wear regular shoes or wear regular-fit long-sleeve shirts. To solve this problem, several measures can be taken, one of which is using thin flaps. However, in cases of large defects, flaps may become limited. In addition, choosing big and bulky flaps might be inevitable to cover large defects, particularly in cases of severe extremity trauma. Primary thinning at the time of flap harvest has been shown to achieve satisfactory results. However, it is technically demanding and requires a long operating time. Furthermore, it increases the risk of flap vascularity-related complications.

In previous reports, debulking surgery was performed at least 6 months after free flap reconstruction. Most cases needed several operations to complete debulking. Although patient satisfaction can be fairly good, these methods are time-consuming. Moreover, the financial burden can be heavy because of repeated admissions and operations.

In 2005, Tsan-Shiun Lin et al. reported that one-stage debulking surgery circumvented multiple operations. Although there were a few cases of partial necrosis on the surface, all cases completed treatment without additional operations.

One-stage debulking surgery shortens the total treatment time. However, an average waiting period of 3.7 months after free flap reconstruction is required until debulking surgery. Especially, del Rio et al. also reported one-stage debulking surgery. In that report, the average time of one-stage debulking surgery after reconstruction was 9.3 months, which is much shorter than multiple debulking surgery procedures. However, it is still a relatively long treatment period in total. In the cases reported in this article, we started early debulking surgery with tangential excision at approximately 1 week after free flap reconstruction when blood supply became stable. Furthermore, we repeated this procedure three to four times and then performed FTSG to complete it. The total debulking procedure took 3–4 weeks after free flap reconstruction to perform FTSG, thus tremendously shortening treatment periods.

There is another reason why we performed repeated debulking instead of one-stage debulking surgery. Such cases are often cases of severe extremity injuries, and therefore, other multiple injuries may exist. The patients are required to stay in the hospital even after free flap surgery. Most cases are related to severe open fractures, and therefore, the infection is the most concerning issue. We usually take patients to the operating theater twice a week for dressing change and perform irrigation under flap and in other deep area not only in the surface. Debulking procedure is performed as a part of this management.

We selected LD flap in all cases because the soft tissue defects were relatively large. LD flap is widely used because of its versatility. It can cover a large area and can easily be harvested owing to little anatomical variations. However, its bulkiness often becomes problematic, particularly when the flap is used around the ankle or foot because the patient cannot wear regular shoes. In cases where the flap is used around the upper limb, bulkiness is esthetically problematic as it is easily visible and patients cannot put their arms through sleeves. Therefore, in such cases, multiple debulking surgeries are necessary.

We decided that performing tangential excision was safe during the early period approximately 1 week after free flap reconstruction for two major reasons. The first reason was the timing of revascularization after free flap reconstruction.

Whether free flap is revascularized after free flap transfer remains controversial. Acland et al. reported the relationship between flap survival and vessel ligation and found that flap necrosis occurred if the pedicle was ligated within 7 days after flap transfer. However, flaps survived completely if not interrupted until 3 weeks [1]. In contrast, Fisher and Wood reported complete necrosis after axial vessel ligation even after 7 months of LD flap [2]. Sadove and Kanter reported no neovascularization at 3 months after free LD flap [3]. Machens et al. clinically showed that perfusion from vascular pedicle persisted even 10 years after free flap [4].

Tsur et al. reported that flap revascularization became stable approximately 7 days after free flap transfer. Neovascularization is important to occur from the bed of the flap and the edge of the wound. However, revascularization from the bed has been reported to be more important [5].

Khoo et al. also reported that flap survival strongly depended on revascularization from the base when it lost pedicle blood supply from 7 to 14 days after free flap transfer [6].

Ozlem et al. found that flap well survived even though its main pedicle was ligated 7 days after free flap in a rat model. They reported that the reason for flap survival was revascularization from the base and edge of the flap [7].

Based on these reports, we performed debulking surgery after 7 days and tangential excision to not interrupt revascularization from the base. The second reason was the anatomy of LD flap vascularization. The vascular pedicle of the LD flap runs deep to the surface of the flap. Thinning was performed from the surface of the flap, and it did not interrupt the main vessel that lined the deep surface. This possibly explains why it might be safe to perform debulking even in the early stage after free flap transfer.

The limitation of this report was the fact that all cases underwent free LD flap and the number of cases was small. Accumulation of more cases is required to confirm the efficacy of this method. Moreover, the same procedure should be performed using other muscle/cutaneous flaps.

## Conclusion

The method we describe in this article could provide not only safe and good results after free LD flap reconstruction for large soft tissue defects but also a complete debulking procedure in the early stage of treatment.

## Ethical approval

This study was approved by the Institutional Review Board of Sapporo Higashi Tokushukai Hospital (approval number: 22_07).

## Consent

Written informed consent was obtained from the patients for the publication of this case report and accompanying images. A copy of the written consent is available for review by the Editor-in-Chief of this journal on request.
